# Redefining evidence for teprotumumab in thyroid eye disease: an updated meta-analysis of efficacy and safety

**DOI:** 10.3389/fendo.2026.1735660

**Published:** 2026-02-13

**Authors:** Rongjing Song, Wei Zhao, Shasha Li, Xiaofang Niu, Jing Guo, Xiuying Zhang, Xiaohong Zhang, Linong Ji

**Affiliations:** 1 Department of Pharmacy, Peking University People’s Hospital, Beijing, China; 2 Department of Endocrinology and Metabolism, Peking University People’s Hospital, Peking University Diabetes Centre, Beijing, China; 3 Department of Clinical Pharmacy, Heze Municipal Hospital, Heze, Shandong, China; 4 Department of Obstetrics and Gynecology, Peking University People’s Hospital, Beijing, China

**Keywords:** teprotumumab, insulin-like growth factor 1 receptor, thyroid eye disease, meta-analysis, efficacy, safety

## Abstract

**Background:**

Thyroid eye disease (TED) is a sight-threatening autoimmune disorder with limited effective therapies. Teprotumumab, an insulin-like growth factor-1 receptor inhibitor, has emerged as a promising treatment. However, a comprehensive synthesis of its efficacy and safety across randomized trials remains limited.

**Methods:**

A systematic review and meta-analysis of randomized controlled trials (RCTs) comparing teprotumumab with placebo in TED was conducted. Primary outcomes included proptosis response, overall response, change in proptosis, diplopia response, achievement of a Clinical Activity Score (CAS) ≤1, changes in Graves’ ophthalmopathy–specific quality-of-life questionnaire (GO-QOL) scores and safety outcomes. Pooled risk ratios (RRs) and mean differences (MDs) with 95% confidence intervals (CIs) were calculated using random-effects models.

**Results:**

Seven RCTs involving 438 participants were included. Teprotumumab significantly improved all efficacy outcomes: proptosis response (RR, 6.87; 95% CI, 3.32 to 14.24), overall response (RR, 7.82; 95% CI, 3.36 to 18.18), reduction in proptosis (MD, -2.46 mm; 95% CI, -2.96 to -1.96), diplopia response (RR, 1.85; 95% CI, 1.28 to 2.68), CAS ≤1 (RR, 3.39; 95% CI, 2.41 to 4.78) and increase in GO-QOL overall score (MD, 10.87; 95% CI, 9.91 to 11.83). Safety analysis indicated elevated risks of hyperglycemia (RR, 2.82; 95% CI, 1.08 to 7.37), muscle spasms (RR, 3.83; 95% CI, 1.97 to 7.43), dry skin (RR, 6.54; 95% CI, 1.52 to 28.09), and hearing impairment (RR, 3.74; 95% CI, 1.26 to 11.13).

**Conclusions:**

Teprotumumab provides substantial, consistent benefits in improving proptosis, diplopia, disease activity and GO-QOL in TED. Clinicians should monitor for adverse events, particularly hyperglycemia and hearing impairment. These findings reinforce teprotumumab as a pivotal therapeutic option and support balanced risk-benefit evaluation.

## Introduction

Thyroid eye disease (TED), also known as Graves’ orbitopathy, is an autoimmune inflammatory disorder of the orbit that is both disfiguring and potentially sight-threatening. It represents the most common orbital disease in adults, with an estimated annual incidence of 16 per 100,000 women and 3 per 100,000 men (1). The disease involves overexpression of insulin-like growth factor 1 receptor (IGF-IR) on orbital fibroblasts and immune cells, with synergistic signaling between IGF-IR and the thyrotropin receptor driving orbital inflammation, fibroblast proliferation, and tissue remodeling (2, 3). These processes lead to proptosis, eyelid retraction, diplopia, and, in severe cases, optic neuropathy and corneal damage, profoundly affecting patients’ quality of life and psychosocial well-being (4). Current first-line therapy for active, moderate-to-severe TED is intravenous methylprednisolone, administered either as monotherapy or, in selected cases, in combination with other immunosuppressive agents. However, their efficacy–particularly for proptosis and diplopia–remains limited, and treatment is frequently complicated by adverse effects and high relapse rates (5, 6). These challenges highlight an unmet need for targeted and effective therapies.

The elucidation of the central role of IGF-IR in TED pathogenesis has paved the way for novel biologic therapies. Teprotumumab, a fully human monoclonal antibody against IGF-IR, represents a major breakthrough in TED treatment. By blocking IGF-IR signaling, it disrupts the inflammatory and pro-fibrotic cascades (3, 7). Early randomized controlled trials (RCTs) demonstrated remarkable efficacy of teprotumumab compared with placebo, showing significantly higher proptosis response rates (≥ 2 mm reduction), greater reductions in Clinical Activity Score (CAS), and substantial improvements in diplopia and Graves’ ophthalmopathy-specific quality-of-life (GO-QOL) scores (8, 9). Based on these pivotal findings, teprotumumab received approval from the U.S. Food and Drug Administration and has been incorporated into international guidelines for the treatment of active TED (10). More recently, trials conducted in Asian populations using an analogous IGF-IR inhibitor (IBI311) have confirmed comparable benefits, with rapid and clinically meaningful reductions in proptosis and disease activity (11, 12).

Despite these compelling results, the broader evidence base for teprotumumab continues to evolve, and several uncertainties remain. While overall efficacy is robust, the magnitude of improvement in diplopia has not been uniformly consistent across all patient subgroups or subsequent trials (12). Furthermore, although the safety profile appears acceptable, emerging post-marketing data have highlighted adverse events of concern, including hearing impairment, hyperglycemia, and muscle spasms (13–16). The publication of new RCTs from diverse ethnic populations and the availability of extended follow-up data provide an opportunity to re-evaluate the totality of evidence (11, 12). Accordingly, we conducted a systematic review and meta-analysis to assess the efficacy and safety of teprotumumab across all available high-quality RCTs, aiming to provide an updated, quantitative synthesis to guide clinical practice and future guideline development in the management of TED.

## Materials and methods

**Figure 1:**
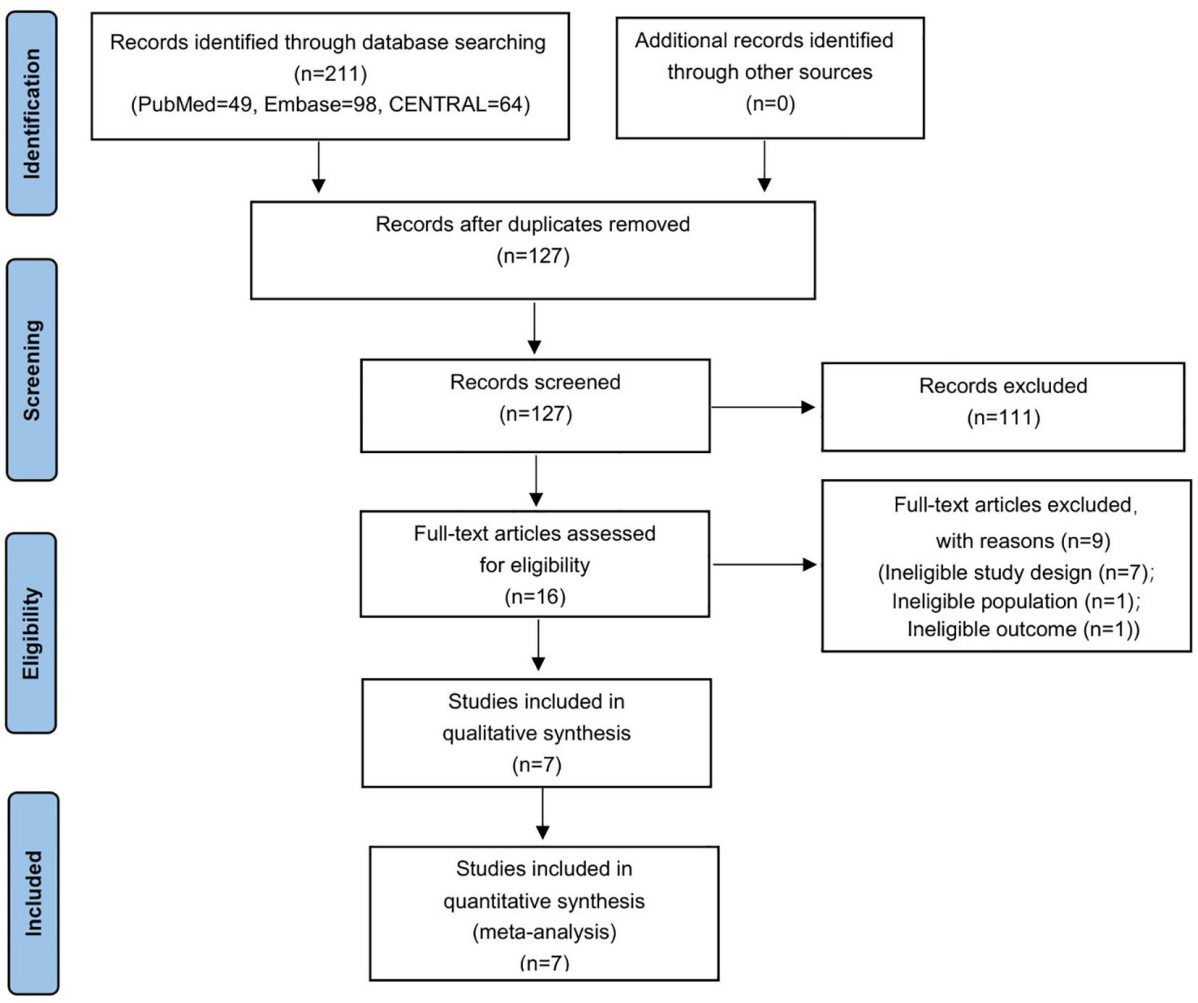
Study selection flowchart.

This meta-analysis was conducted and reported in accordance with the Preferred Reporting Items for Systematic Reviews and Meta-Analyses (PRISMA) guideline. The corresponding PRISMA flow diagram is shown in **Figure 1**.

### Inclusion and exclusion criteria

Studies were eligible for inclusion if they met the following criteria: 1) participants were diagnosed with TED; 2) the intervention group received eight infusions of teprotumumab (10 mg/kg for the first infusion and 20 mg/kg for each of the remaining seven infusions) administered once every three weeks; 3) primary and secondary end points reported in the trials included proptosis response rate (defined as a reduction in proptosis of ≥2 mm from baseline at week 24), overall response rate (defined as a composite of a ≥ 2 mm reduction in proptosis and ≥2-point reduction in CAS), change in proptosis from the baseline, diplopia response rate (defined as a reduction in diplopia of ≥1 grade from baseline according to the Gorman subjective diplopia score at week 24) (2) among patients with diplopia at baseline, the proportion of subjects achieving a CAS of 0/1 at week 24, change in GO-QOL score from baseline; 4) the study design was a randomized control trial; 5) the language used for the study was either English or Chinese; and 6) the full text was available online. Retrospective cohort studies, systematic reviews, case reports, studies without human data, conference abstracts and single-arm trials were excluded.

### Literature search and study selection

A systematic literature search was conducted in PubMed, EMBASE, and the Cochrane Central Register of Controlled Trials (CENTRAL) from database inception to October 2025. The search strategy combined the following key terms: *thyroid eye disease*, *thyroid-associated ophthalmopathy*, *Graves’ orbitopathy*, *teprotumumab*, and *randomized controlled trial*. Additional relevant studies were identified through manual searches of Google Scholar and other sources. The detailed search strategy is provided in **Supplementary Table S1**. Study selection was performed independently by two investigators according to the predefined eligibility criteria. Discrepancies were resolved by discussion and, when necessary, adjudicated by a third reviewer.

### Assessment of risk of bias and data collection

Two reviewers independently assessed the methodological quality of each included study using the risk of bias tool outlined in the *Cochrane Handbook for Systematic Reviews of Interventions*. The following domains were evaluated: random sequence generation, allocation concealment, blinding of participants and personnel, blinding of outcome assessment, completeness of outcome data, selective reporting, and other potential sources of bias. Each domain was classified as having a low, high, or unclear risk of bias, and an overall judgment was summarized across domains. Data regarding baseline characteristics and all relevant outcomes were extracted in accordance with the predefined criteria. Any discrepancies between reviewers were resolved through consensus discussion.

### Statistical analysis

Statistical analyses were performed using Review Manager (version 5.3). Data were synthesized according to outcome type. Dichotomous outcomes were reported as risk ratios (RRs) with 95% confidence intervals (CIs), and continuous outcomes were reported as mean differences (MDs) with 95% CIs. The inverse variance method was applied for continuous outcomes, and the Mantel–Haenszel method for dichotomous outcomes. Heterogeneity among studies was assessed using the *I²* statistic, with values > 50% indicating substantial heterogeneity. A random-effects model was used for all pooled analyses. A two-sided *p*-value < 0.05 was considered statistically significant.

## Results

### Literature search and study selection

The detailed search strategy is presented in the **Supplementary Table S1**, and the study selection process is summarized in **Figure 1**. A total of 211 studies were identified through database searches (PubMed = 49, EMBASE = 98, and CENTRAL = 64) with no additional studies retrieved from other sources. After removal of duplicates, 127 unique records were screened by title and abstract, of which 111 were excluded. Sixteen full-text articles were subsequently assessed for eligibility, and nine were excluded with documented reasons (ineligible study design, ineligible population or ineligible outcome). Ultimately, seven randomized controlled trials met the inclusion criteria and were included in the meta-analysis, comprising 438 participants in total (teprotumumab, n = 240; placebo, n = 198) (2, 6, 8, 11, 12, 17, 18).

### Study characteristics and data extraction

The characteristics of the studies included in this meta-analysis are summarized in **Table 1**. The publications ranged from 2017 to 2025, and all were multicenter randomized controlled trials conducted across Asia, Europe, and the Americas. Sample sizes per study ranged from 10 to 54 participants per treatment arm. The enrolled populations consisted predominantly of adults, with no significant sex differences reported. Most studies included patients with a CAS greater than 3 at baseline except for the trial by Douglas et al. (6). The duration of TED ranged from 3.4 to 64.8 months, and mean baseline proptosis ranged between 20.4 mm and 24.6 mm. In most trials, the proportion of patients presenting with baseline diplopia was slightly higher in the teprotumumab group than in the placebo group. All studies administered eight intravenous infusions of teprotumumab in the intervention group and reported a follow-up period of 24 weeks. Relevant data were extracted and categorized according to the efficacy and safety profiles of teprotumumab. Efficacy outcomes comprised proptosis response rate, overall response rate, change in proptosis from baseline (mm), diplopia response rate, the proportion of patients achieving a CAS of 0 or 1, change in GO-QOL score from baseline. Safety outcomes were obtained from the included studies, defined as: ‘Muscle spasm’, ‘Alopecia’, ‘Nausea’, ‘Fatigue’, ‘Diarrhea’, ‘Headache’, ‘Dry skin’, ‘Dysgeusia’, ‘Stomatitis’ (or ‘Noninfective gingivitis’), ‘Hearing impairment’ (or ‘Hypoacusis’), ‘Hyperglycemia’ (or ‘Diabetes’, ‘Diabetes mellitus’), ‘Infusion reaction’.

**Table 1:** Characteristics of the studies included in the meta-analysis.

First author/publication year	Study design	Single vs multicenter	Country	Treatment groups (patients, n)	Inclusion criteria		Baseline characteristics (Teprotumumab/placebo)						Interventions	Follow-up duration (weeks)
					Age range (y)	CAS	Age (y)	Female (n)	Smokers (n)	Duration of GO (months)	Baseline proptosis (mm)	Diplopia at baseline (n)		
Smith et al, 2017 (8)	RCT	multicenter	Germany, Italy, United Kingdom, USA	Teprotumumab (42) versus placebo (45)	18-75	≥4/7	52/54	28/36	11/18	10.7/10.8	23.4/23.1	38/31	Eight infusions of teprotumumab*	24
Douglas et al, 2020 (2)	RCT	multicenter	Germany, Italy, USA	Teprotumumab (41) versus placebo (42)	18-80	≥4/7	52/49	29/31	9/8	6.2/6.4	22.6/23.2	28/28	Eight infusions of teprotumumab*	24
Hiromatsu et al, 2025 (11)	RCT	multicenter	Japan	Teprotumumab (27) versus placebo (27)	20-80	≥3/7	47/50	18/20	4/4	4.2/5.2	21.1/20.4	22/20	Eight infusions of teprotumumab*	24
Douglas et al, 2023 (6)	RCT	multicenter	USA	Teprotumumab (42) versus placebo (20)	≥18	≤1/7	49/49	32/18	6/2	64.8/61.2	24.6/24.0	14/4	Eight infusions of teprotumumab*	24
Ugradar et al, 2022–1 (17)	RCT	multicenter	Germany, Italy, United Kingdom, USA	Teprotumumab (10) versus placebo (12)	18-80	>3/7	50/59	6/10	2/4	5.8/6.0	24.2/22.8	10/8	Eight infusions of teprotumumab*	24
Ugradar et al, 2022–2 (18)	RCT	multicenter	Germany, Italy, United Kingdom, USA	Teprotumumab (24) versus placebo (24)	18-80	≥4/7	49/50	14/14	6/4	6.1/6.4	22.2/22.7	22/15	Eight infusions of teprotumumab*	24
Zhang et al, 2025 (12)	RCT	multicenter	China	Teprotumumab (54) versus placebo (28)	18-80	≥3/7	40/39	36/20	11/5	3.9/3.4	20.5/20.4	30/15	Eight infusions of teprotumumab*	24

CAS, clinical activity score; GO, thyroid eye disease; USA, United States of America.

*Eight infusions of teprotumumab: Eight infusions of teprotumumab 10 mg/kg for the first infusion and 20 mg/kg for the remaining seven infusions or placebo, one every three weeks.

Additionally, for change in proptosis from baseline (2, 6, 8, 12) and change in GO-QOL score from baseline (2, 12), the outcomes were presented as mean ± standard error (SEM), the formula Standard Deviation (SD) = SEM × √N was used to obtain the SD. In Hiromatsu et al. (11), change in proptosis from baseline and change in GO-QOL score from baseline were presented as 95% CI, and the corresponding SD was calculated using the built-in calculator in Review Manager (version 5.3) by entering the mean, sample size, and 95% CI. For dichotomous outcomes, the proportion of subjects with a CAS ≤ 1 was reported as percentages in Smith et al. (8), and we obtained the corresponding numerical data through simple multiplication. Two researchers independently perform data extraction and conversion, with any disagreements resolved through discussion or consultation with a third researcher.

### Assessment of risk of bias

**Figure 2:**
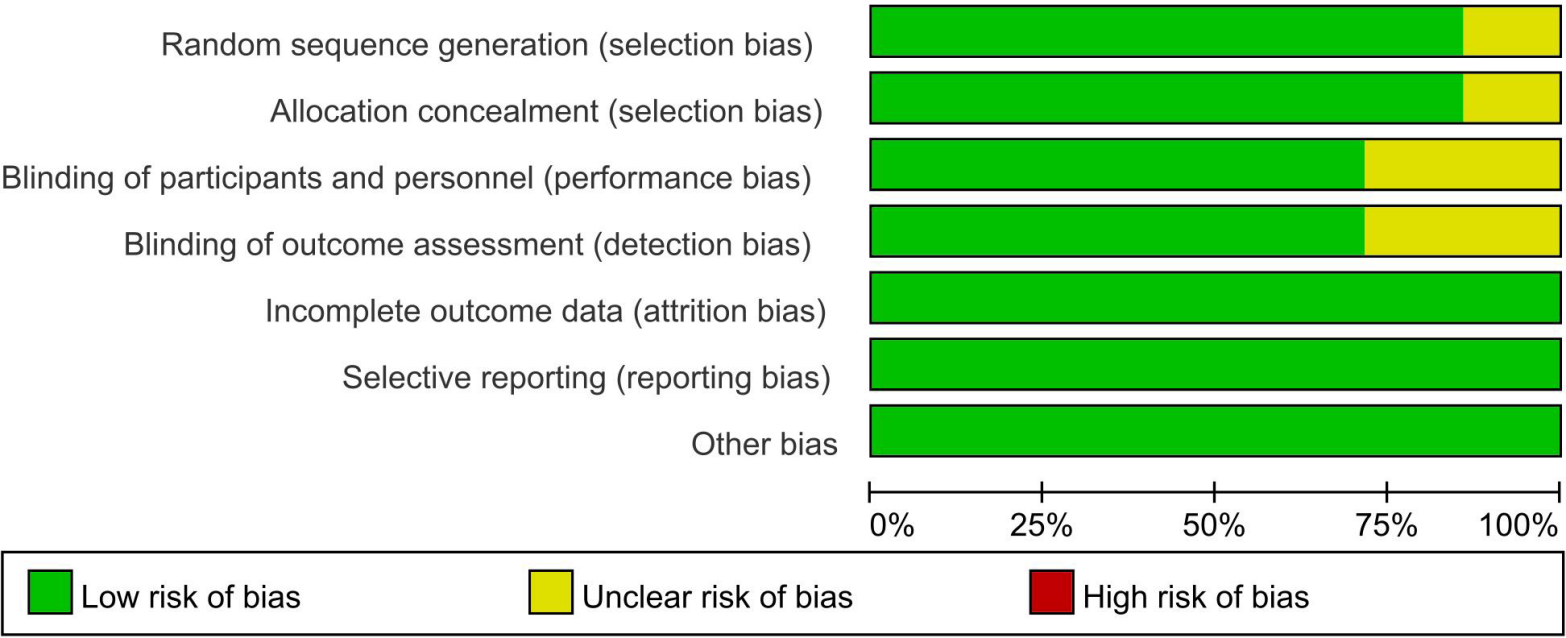
Risk of bias of studies comparing teprotumumab and placebo.

**Figure 3:**
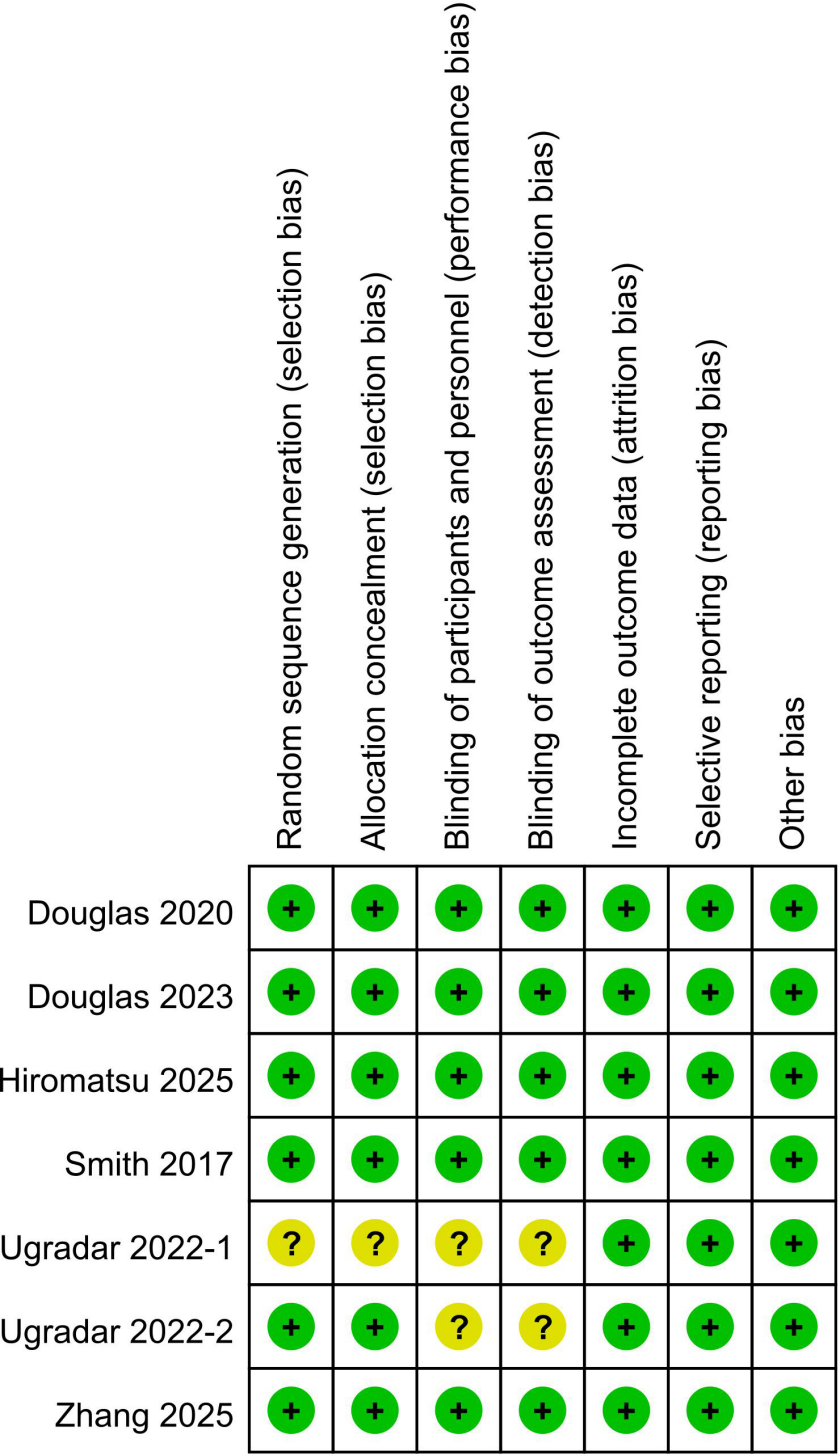
Risk of bias summary of studies comparing teprotumumab and placebo.

A methodological quality assessment was conducted for all seven included studies. **Figure 2** depicts the risk of bias for each individual studies comparing teprotumumab with placebo, and **Figure 3** presents a summary of these findings. Six studies explicitly reported adequate methods for random sequence generation and allocation concealment (2, 6, 8, 11, 12, 18), while one study did not provide sufficient details in these domains (17). With respect to blinding, five studies were judged to have a low risk of bias for both participants/personnel and outcome assessment (2, 6, 8, 11, 12), while two were rated as having an unclear risk (17, 18). No evidence of attrition bias or selective reporting bias was detected in any of the included studies (2, 6, 8, 11, 12, 17, 18), and no other substantial sources of bias were identified. Overall, the majority of the data synthesized in this meta-analysis originated from studies with a low risk of bias.

### Meta-analysis for the efficacy of teprotumumab

**Figure 4:**
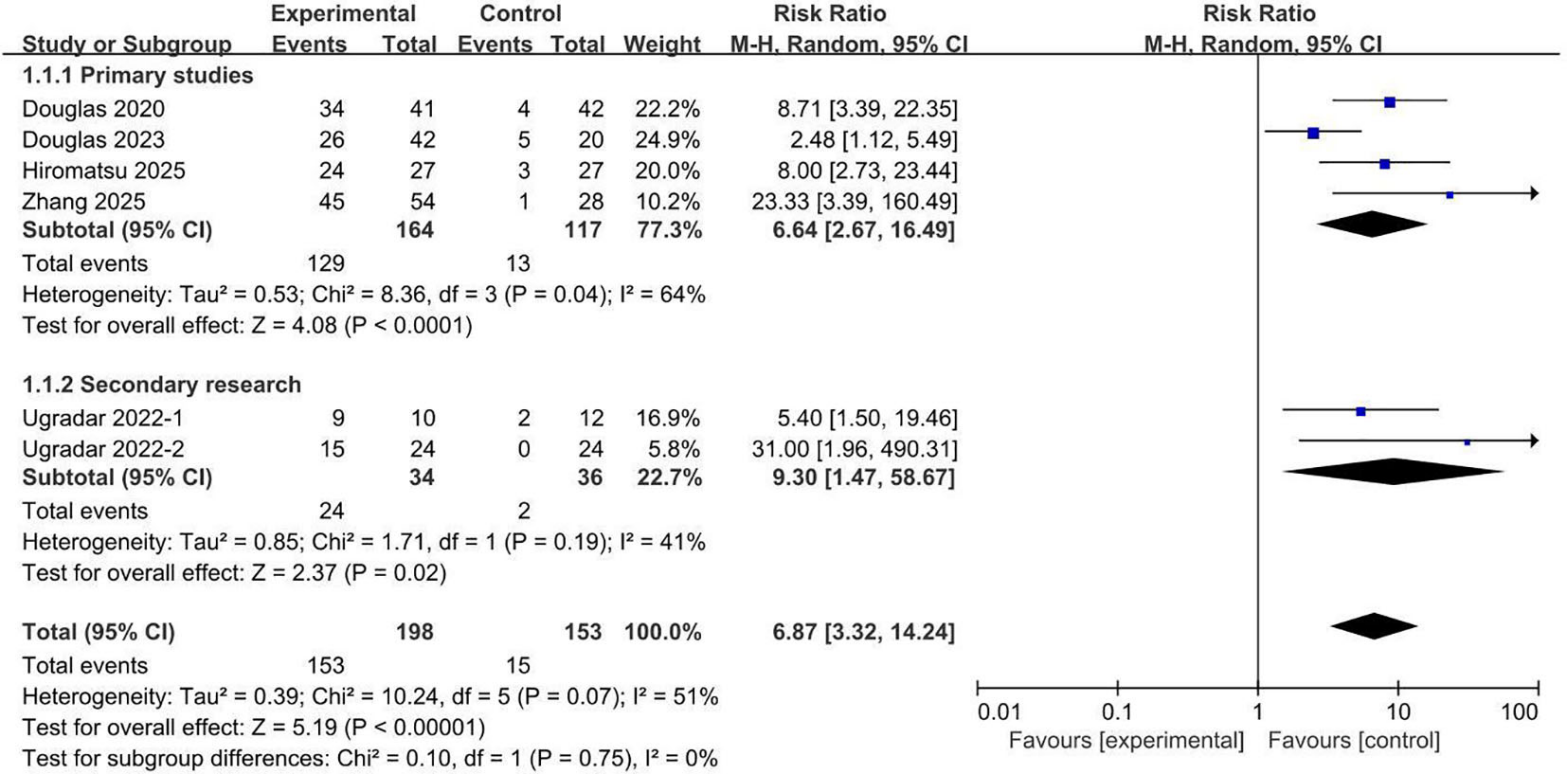
Proptosis response rate comparing teprotumumab and placebo.

Six studies reported data on the proptosis response rate. At the predefined 24-week endpoint, teprotumumab was associated with a significantly higher likelihood of achieving a proptosis response rate compared to placebo (**Figure 4**). The pooled RR for response was 6.87 (95% CI, 3.32 to 14.24; *P* < 0.00001). Analyses stratified by trial type produced consistent findings: the four primary trials demonstrated a RR of 6.64 (95% CI, 2.67 to 16.49; *P* < 0.0001) (2, 6, 11, 12), and the two secondary trials yielded a RR of 9.30 (95% CI, 1.47 to 58.67; *P* = 0.02) (17, 18). Collectively, these results provide robust evidence that teprotumumab is highly effective in achieving a proptosis response in patients with TED.

**Figure 5:**
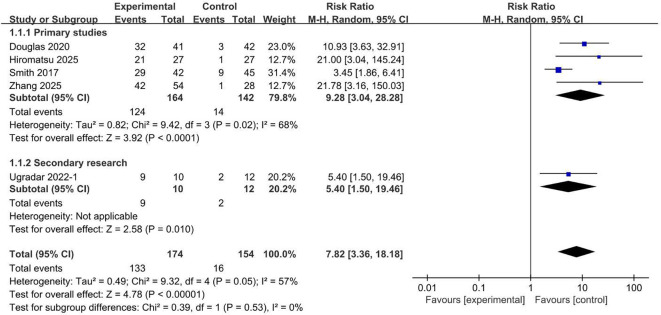
Overall response rate comparing teprotumumab and placebo.

The overall response was evaluated in five included studies. Pooled analysis demonstrated that teprotumumab was associated with a significantly higher overall response compared to placebo (**Figure 5**), with a RR of 7.82 (95% CI, 3.36 to 18.18; *P* < 0.00001). This treatment effect was consistent across predefined trial types, including both the primary studies (RR, 9.28; 95% CI, 3.04 to 28.28; *P* < 0.0001) and the secondary studies (RR, 5.40; 95% CI, 1.50 to 19.46; *P* = 0.01) (17). These findings reinforce the robust efficacy of teprotumumab in improving the overall therapeutic response of TED.

**Figure 6:**
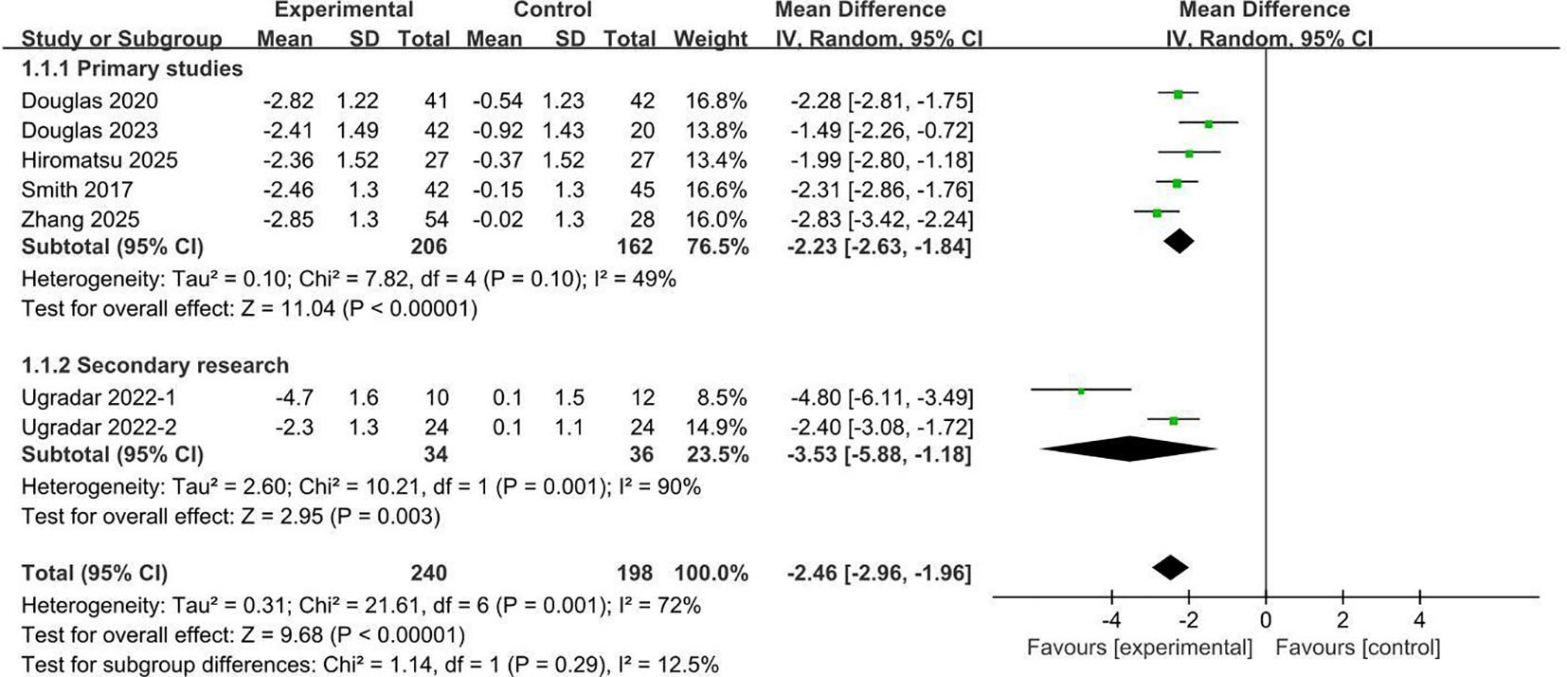
Change in proptosis from the baseline comparing teprotumumab and placebo.

All seven studies reported the mean change in proptosis from baseline. Meta-analysis confirmed that teprotumumab produced a significantly greater reduction in proptosis compared with placebo (**Figure 6**). Treatment with teprotumumab resulted in a pronounced decrease in proptosis, whereas a slight increase was observed in the placebo groups. The pooled MD was -2.46 mm (95% CI, -2.96 to -1.96; *P* < 0.00001). This therapeutic benefit was consistently observed in analyses stratified by trial type of the primary studies (MD, -2.23 mm; 95% CI, -2.63 to -1.84; *P* < 0.00001) and the secondary analyses (MD, -3.53 mm; 95% CI, -5.88 to -1.18; *P* = 0.003). Collectively, these findings confirm that teprotumumab provides a significant and clinically meaningful improvement in proptosis in TED.

**Figure 7:**
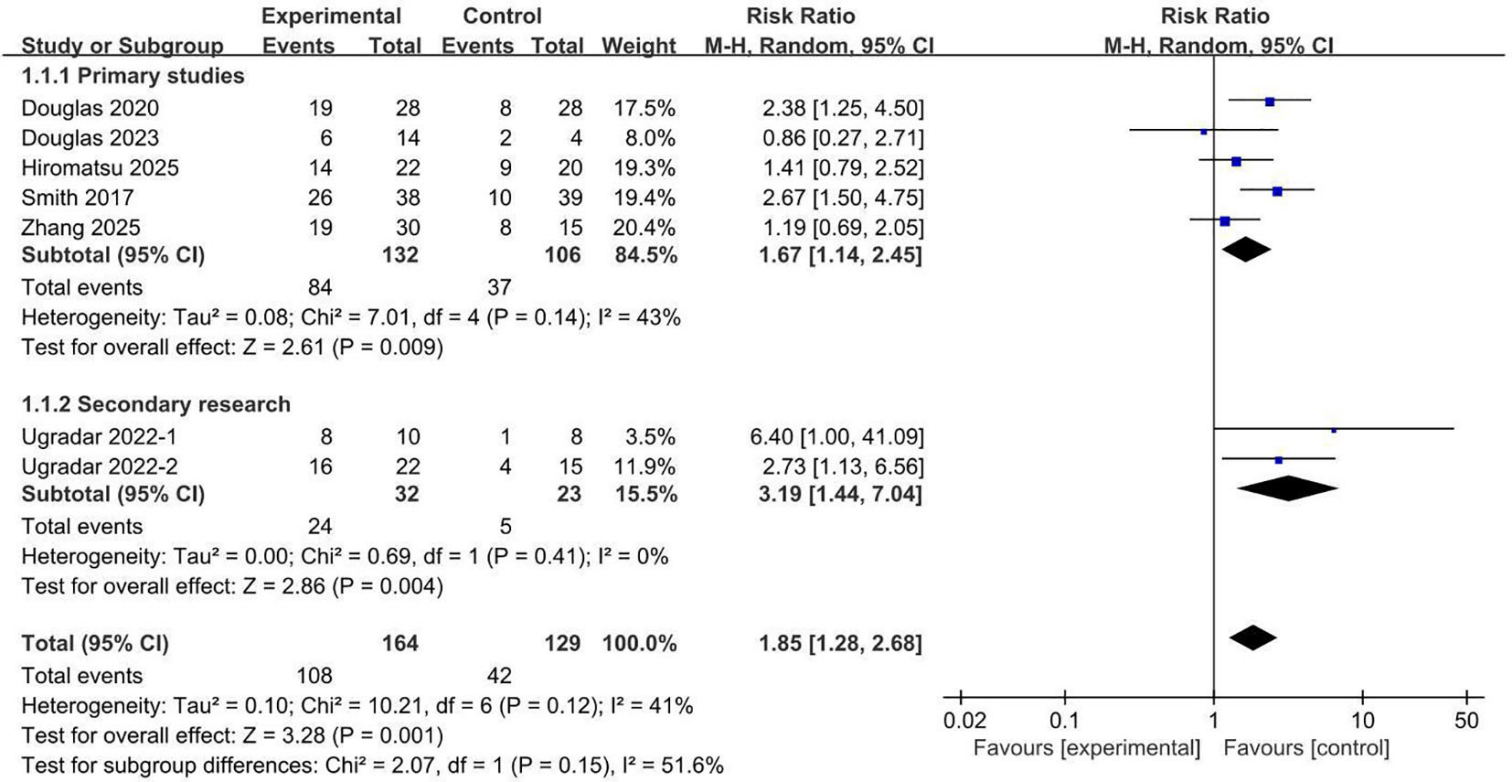
Diplopia response rate comparing teprotumumab and placebo.

Data on the diplopia response rate were available from all seven studies. Six of these trials demonstrated a consistent benefit favoring teprotumumab, with only the study by Douglas et al. (6) serving as an outlier. The pooled analysis revealed a statistically significant improvement in diplopia response with teprotumumab compared placebo (**Figure 7**), with a RR of 1.85 (95% CI, 1.28 to 2.68; p = 0.001). This primary finding was further supported by analyses stratified by trial type, which yielded concordant and statistically significant results (RR, 1.67; 95% CI, 1.14 to 2.45; *P* = 0.009; and RR, 3.19; 95% CI, 1.44 to 7.04; *P* = 0.004). Together, these results provide robust evidence that teprotumumab effectively improves diplopia in patients with TED.

**Figure 8:**
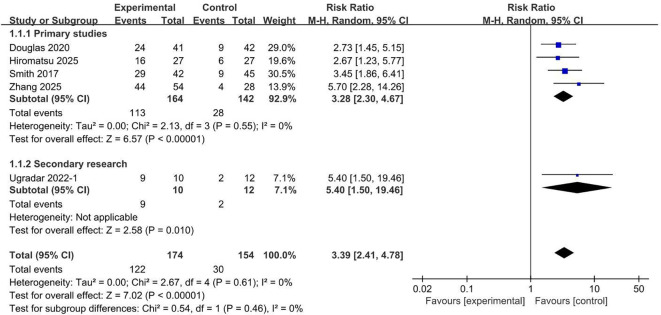
Proportion of subjects with a clinical activity score ≤1 comparing teprotumumab and placebo.

Teprotumumab was associated with a significantly higher likelihood of achieving a CAS of ≤1 compared with placebo based on a meta-analysis of five studies (2, 8, 11, 12, 17) (**Figure 8**), reaching a pooled RR of 3.39 (95% CI, 2.41 to 4.78; *P* < 0.00001). This treatment effect was consistently observed across predefined trial types, including primary studies (RR, 3.28; 95% CI, 2.30 to 4.67; *P* < 0.00001) and secondary studies (RR, 5.40; 95% CI, 1.50 to 19.46; *P* = 0.01). Collectively, these findings confirm that teprotumumab significantly reduces inflammatory disease activity in TED.

**Figure 9:**
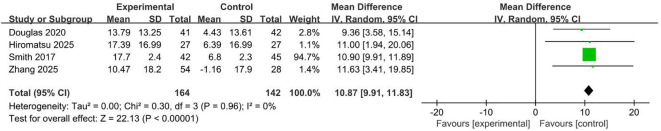
Change in GO-QOL score from baseline comparing teprotumumab and placebo.

Four studies reported the mean change in GO-QOL overall score from baseline. Meta-analysis confirmed that the changes in GO-QOL scores from the baseline to week 24 were greater in the teprotumumab group compared to the placebo group (**Figure 9**). The pooled MD was 10.87 points (95% CI, 9.91 to 11.83; P < 0.00001). These findings confirm that teprotumumab provides a significant meaningful improvement in GO-QOL in TED.

Furthermore, trial-level analyses stratified by study region (Europe/America vs. Asia) were performed, with results presented in the **Supplementary Figures S1**–**S6**. Across regions, teprotumumab consistently demonstrated strong efficacy in achieving a proptosis response, improving the overall therapeutic response, and providing a statistically and clinically meaningful reduction in proptosis. Improvements in diplopia were observed across regions; however, in the Asian subgroup, the improvement in diplopia did not reach statistical significance. Teprotumumab was also associated with a significantly higher likelihood of achieving a CAS of ≤1 compared with placebo, as well as a statistically and clinically meaningful improvement in the GO−QOL scores.

### Meta-analysis for the safety of teprotumumab

**Figure 10:**
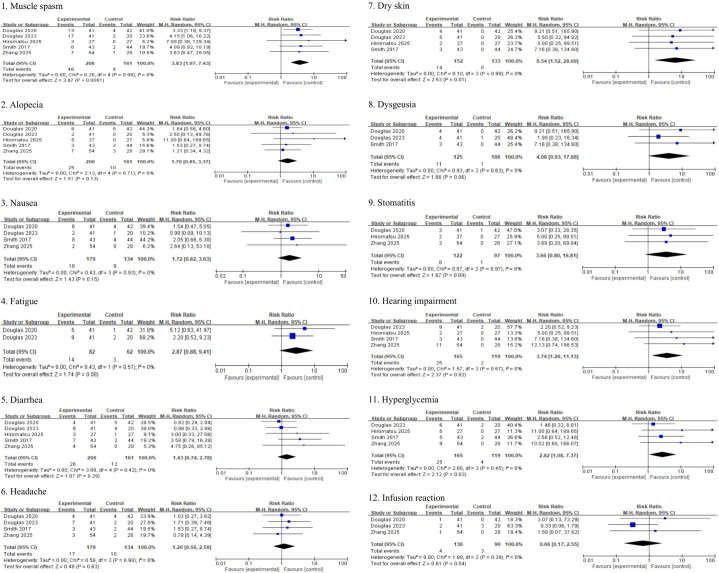
Adverse drug reaction comparing teprotumumab and placebo.

**Figure 10** presents the pooled analysis of adverse events comparing teprotumumab with placebo. Gastrointestinal events, including nausea (RR, 1.72; 95% CI, 0.82 to 3.63; *P* = 0.15), diarrhea (RR, 1.43; 95% CI, 0.74 to 2.78; *P* = 0.29), and stomatitis (RR, 3.66; 95% CI, 0.80 to 16.81; *P* = 0.09), were numerically more frequent in the teprotumumab group but did not reach statistically significance. Similarly, non-significant increases were observed for fatigue (RR, 2.87; 95% CI, 0.88 to 9.41; *P* = 0.08), alopecia (RR, 1.70; 95% CI, 0.85 to 3.37; *P* = 0.13), and dysgeusia (RR, 4.08; 95% CI, 0.93 to 17.88; *P* = 0.06). In contrast, teprotumumab treatment was associated with a statistically increased risk of hyperglycemia (RR, 2.82; 95% CI, 1.08 to 7.37; *P* = 0.03], muscle spasms (RR, 3.83; 95% CI, 1.97 to 7.43; *P* < 0.0001), dry skin (RR, 6.54; 95% CI, 1.52 to 28.09; *P* = 0.01), and hearing impairment (RR, 3.74; 95% CI, 1.26 to 11.13; *P* = 0.02) compared to placebo. No significant differences between treatment groups were observed for headache (RR, 1.20; 95% CI, 0.56 to 2.59; *P* = 0.63) or infusion-related reactions (RR, 0.66; 95% CI, 0.17 to 2.55; *P* = 0.54).

### Heterogeneity and sensitivity analysis

To assess the robustness of the primary outcomes, a sensitivity analysis was performed after excluding the trial by Douglas et al. (6), which was the only study to include patients in the inactive phase of disease (CAS < 3). As summarized in **Table 2**, the exclusion of this study did not materially alter the results. Teprotumumab remained associated with significantly greater efficacy than placebo across all endpoints, including proptosis response, diplopia response, and the mean change in proptosis from baseline (all *P* < 0.00001). Moreover, this exclusion resulted in a substantial reduction in the *I²* statistic, indicating that the inclusion of participants with inactive disease was a principal source of heterogeneity in the overall analysis.

**Table 2:** Summary of sensitivity analysis with one study removed.

Outcomes	No. of studies	No. of patients in teprotumumab group	No. of patients in control group	Mean difference (95% Cl)	Relative risk (95% Cl)	P value	I^2^ value
Proptosis Response	5	156	133	–	8.91 [5.00, 15.86]	<0.00001	0%
Diplopia Response	6	150	125	–	1.97 [1.36, 2.86]	0.0004	41%
Change in proptosis from baseline	6	198	178	-2.60 [-3.10, -2.10]	–	<0.00001	68%

Cl, confidence interval; I^2^, heterogeneity.

## Discussion

This systematic review and updated meta-analysis, encompassing seven RCTs with a total of 438 patients, provides a comprehensive evaluation of the efficacy and safety of teprotumumab for TED. The pooled evidence demonstrates that teprotumumab confers significantly greater therapeutic benefit than placebo across all prespecified efficacy endpoints, including proptosis response, overall response, mean reduction in proptosis, improvement in diplopia, change in GO-QOL overall scores and achievement of a CAS of 0 or 1. Consistent results were observed in trial-level analyses stratified by study region, further supporting the robustness of these findings. In parallel, the safety analysis revealed a generally manageable adverse event profile, although the risks of hyperglycemia, muscle spasms, and hearing impairment were significantly higher in the teprotumumab group. The consistently low risk of bias across the included trials supports the robustness and reliability of these pooled estimates.

The profound efficacy of teprotumumab demonstrated in this meta-analysis reinforces the findings of the landmark trials that established its clinical role. The marked proptosis response and significant mean reduction in proptosis are consistent with the proposed mechanistic of IGF-1R inhibition, which directly mitigates orbital tissue remodeling (2, 8, 19). These results are concordant with the pivotal trials by Smith et al. and with previous meta-analyses reporting similar magnitudes of benefit (2, 8, 20, 21). Importantly, our pooled analysis clarifies previous inconsistencies regarding diplopia improvement. While the recent RESTORE-1 trial (evaluating IBI311) did not show a statistically significant effect on diplopia, this is likely attributable to a lower baseline prevalence and different severity of diplopia in its cohort, which limited statistical power (12). By integrating data across a larger aggregate sample, our analysis provides a more precise and stable estimate, confirming that teprotumumab confers a statistically and clinically meaningful improvement in this functionally critical endpoint among patients with TED.

An important insight from this meta-analysis concerns the exploration of heterogeneity. The substantial heterogeneity (*I²* > 50%) observed in the initial analyses for several efficacy outcomes was markedly reduced after exclusion of the trial by Douglas et al. (6) in the sensitivity analysis. This study was uniquely enrolled patients with chronic, low-activity TED (CAS ≤ 1), a population that is both phenotypically and pathophysiologically distinct from the active TED patients included in the other trials (22). The marked reduction in *I²* identifies disease activity status as a major source of heterogeneity. Notably, exclusion of this trial did not affect the statistical significance of any efficacy outcome, underscoring the robustness of our primary findings. These results suggest that while the magnitude of benefit may vary with disease activity, teprotumumab retains measurable efficacy not only in active TED but potentially also in patients with chronic, inactive disease–possibly through attenuation of fibrotic remodeling (6, 23). Furthermore, analyses stratified by trial type differentiating primary interventional RCTs from smaller, secondary analyses (17, 18) demonstrated concordant directionality and significance of treatment effects, reinforcing the generalizability of teprotumumab’s efficacy beyond the narrowly defined populations of the pivotal trials.

Several patient-related factors, including smoking status and age, are known to influence disease activity, severity, and therapeutic response in thyroid eye disease (24, 25). However, none of the included randomized controlled trials reported treatment effects stratified by smoking status or age, precluding patient-level subgroup analyses. Nevertheless, baseline characteristics across the included studies were broadly comparable, with similar age distributions and smoking proportions between treatment arms within individual trials, suggesting that these factors were unlikely to have substantially confounded the pooled estimates. Future studies and individual patient data meta-analyses are warranted to further clarify the influence of these factors on response to teprotumumab.

The safety profile elucidated in this meta-analysis underscores the need for careful risk-benefit evaluation and proactive monitoring. Several known adverse drug reactions consistent with the pharmacologic mechanism of IGF-1R inhibition were confirmed. The significantly increased risks of muscle spasms and hyperglycemia are well-documented in prior studies (9, 13). Reported hyperglycemia events ranged from transient, asymptomatic elevations in blood glucose to new-onset insulin-requiring diabetes (2, 13, 26). Mechanistically, teprotumumab-induced IGF-1R blockade disrupts glucose homeostasis by enhancing growth hormone-mediated insulin resistance and impairing insulin signaling through internalization of IGF-1R/insulin receptor hybrid complexes. Consequently, baseline assessment of glycemic status and regular glucose monitoring throughout treatment are strongly recommended to mitigate the risk of hyperglycemia (27–30).

Of particular clinical importance is hearing impairment, which showed a 3.74-fold increased risk compared with placebo. This otologic adverse event–manifesting as tinnitus, ear fullness, or autophony–has been increasingly characterized in recent prospective investigations (14, 16). Although often reversible following dose interruption or treatment completion, these effects can be distressing and warrant pre-treatment audiometric assessment and thorough patient counseling (15). Other adverse events, such as gastrointestinal disturbances, occurred more frequently in the teprotumumab group but were typically mild to moderate in severity.

### Limitations in current evidence

Despite its strengths, this study has several limitations. First, the relatively short follow-up duration (24 weeks) of the included RCTs limits the ability to draw conclusions regarding the long-term durability of response, relapse rates, and the emergence of late-onset adverse events. Second, the modest sample size for some rare adverse events may have resulted in imprecise risk estimates. Finally, the absence of individual patient data precluded a more detailed exploration of patient-specific modifiers of treatment response–such as thyroid function status, prior therapies, and disease duration–which may influence clinical outcomes.

### Conclusions

This meta-analysis provides high-level evidence that teprotumumab offers substantial clinical benefits for patients with thyroid eye disease, effectively improving the disabling manifestations of proptosis and diplopia. The consistency of these findings across diverse study populations supports the robustness of the therapeutic effect. At the same time, teprotumumab is associated with a higher incidence of specific adverse events–most notably hyperglycemia, muscle spasms, and hearing impairment-which are generally manageable with appropriate monitoring. Its clinical use should therefore be accompanied by proactive risk assessment and vigilant monitoring strategies. Future research should refine patient selection, establish long-term efficacy and retreatment frameworks, and develop management protocols to optimize the therapeutic value of this targeted IGF-1R inhibitor.

## Data Availability

The original contributions presented in the study are included in the article/**Supplementary Material**. Further inquiries can be directed to the corresponding authors.

## Supplementary material

Supplementary Figure 1 Proptosis response rate comparing teprotumumab and placebo.

Supplementary Figure 2 Overall response rate comparing teprotumumab and placebo.

Supplementary Figure 3 Change in proptosis from the baseline comparing teprotumumab and placebo.

Supplementary Figure 4 Diplopia response rate comparing teprotumumab and placebo.

Supplementary Figure 5 Proportion of subjects with a clinical activity score ≤1 comparing teprotumumab and placebo.

Supplementary Figure 6 Change in GO-QOL score from baseline comparing teprotumumab and placebo.
