# Supplementary figures and images for: Redefining evidence for teprotumumab in thyroid eye disease: an updated meta-analysis of efficacy and safety
Source: Front Endocrinol (Lausanne). 2026 Feb 13;17:1735660. doi: 10.3389/fendo.2026.1735660 (PMC12945760; doi:10.3389/fendo.2026.1735660)

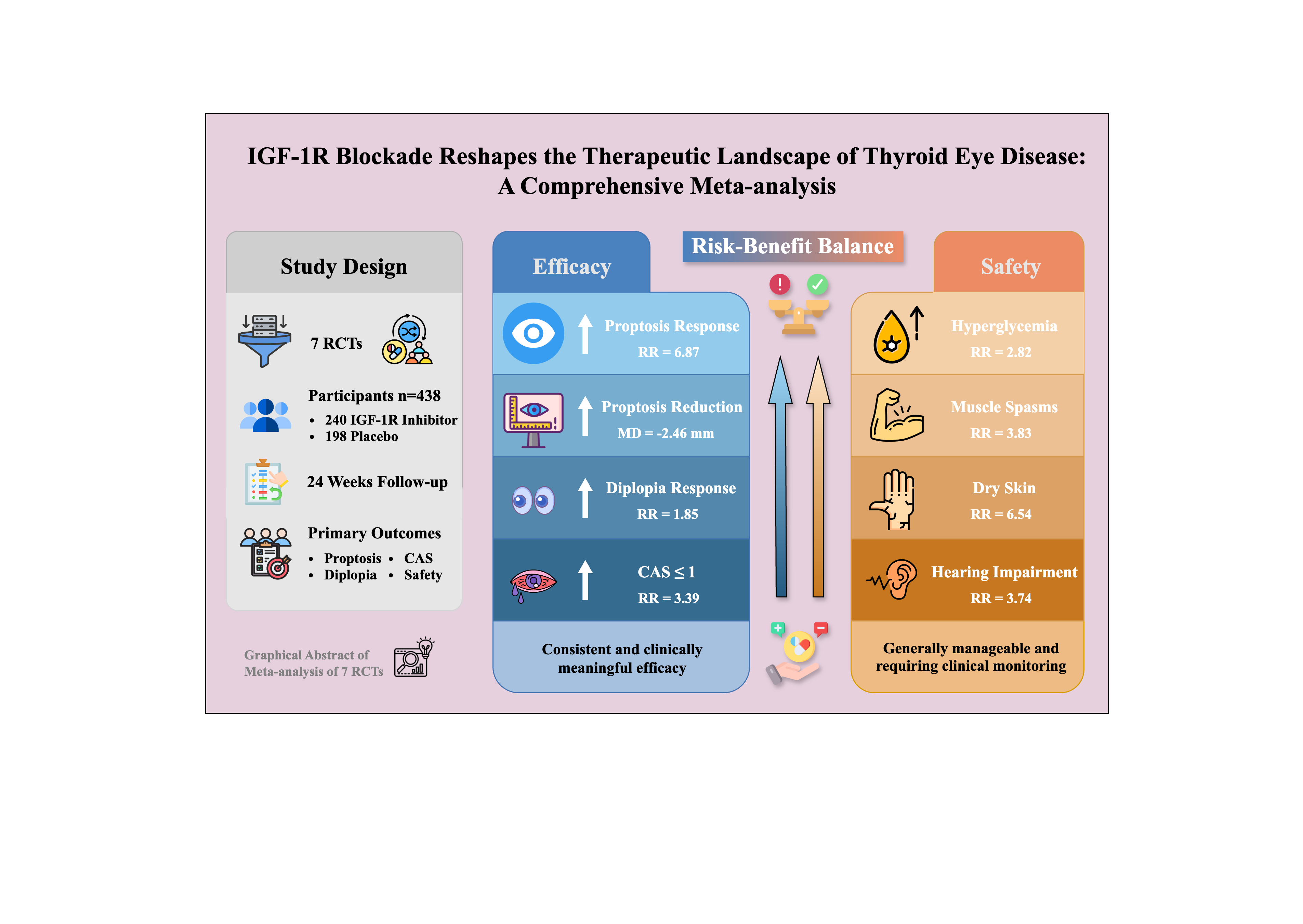

Supplement: Supplementary Figure 1 — Proptosis response rate comparing teprotumumab and placebo. [file Image1.jpeg]

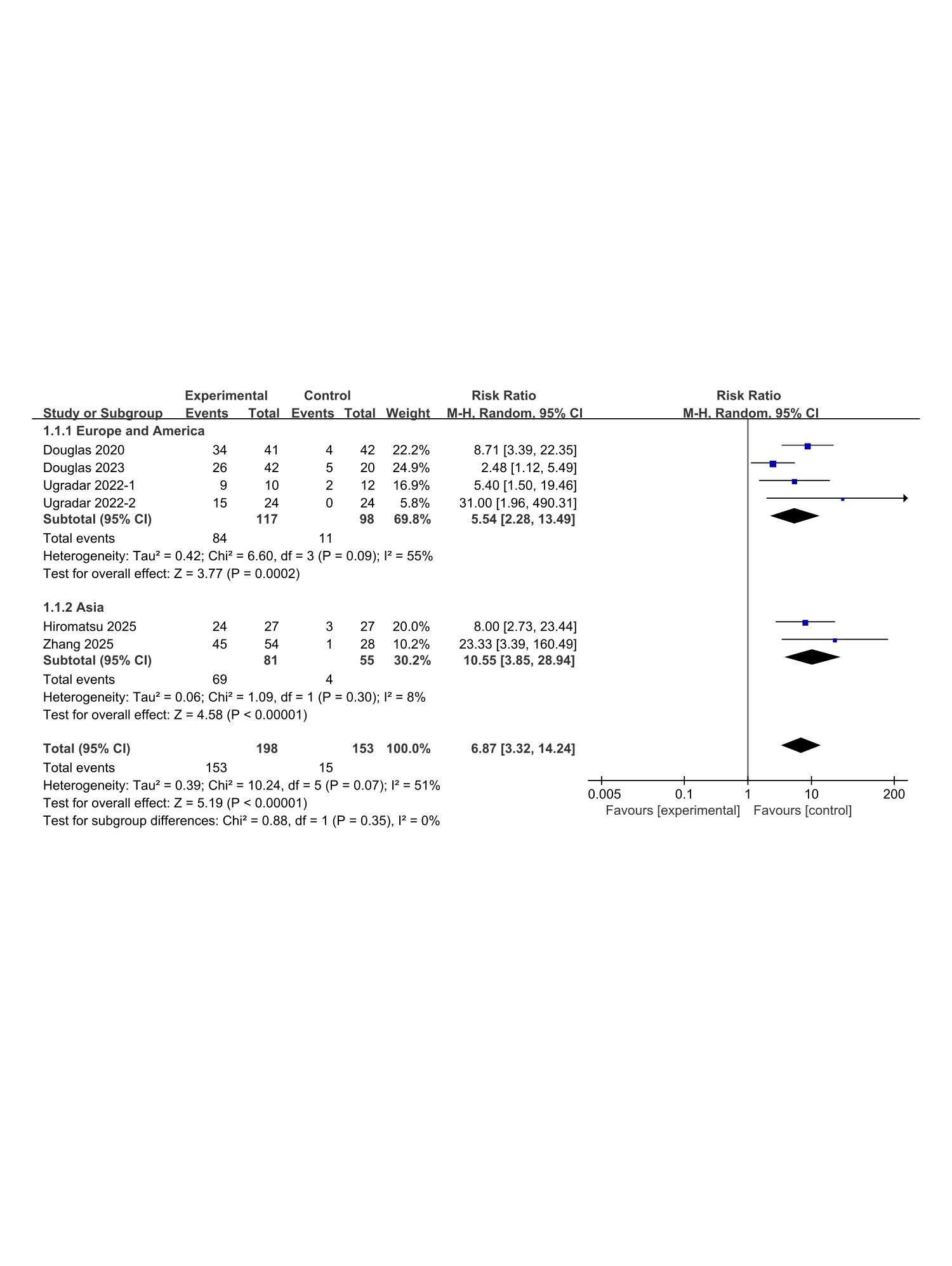

Supplement: Supplementary Figure 2 — Overall response rate comparing teprotumumab and placebo. [file Image2.jpeg]

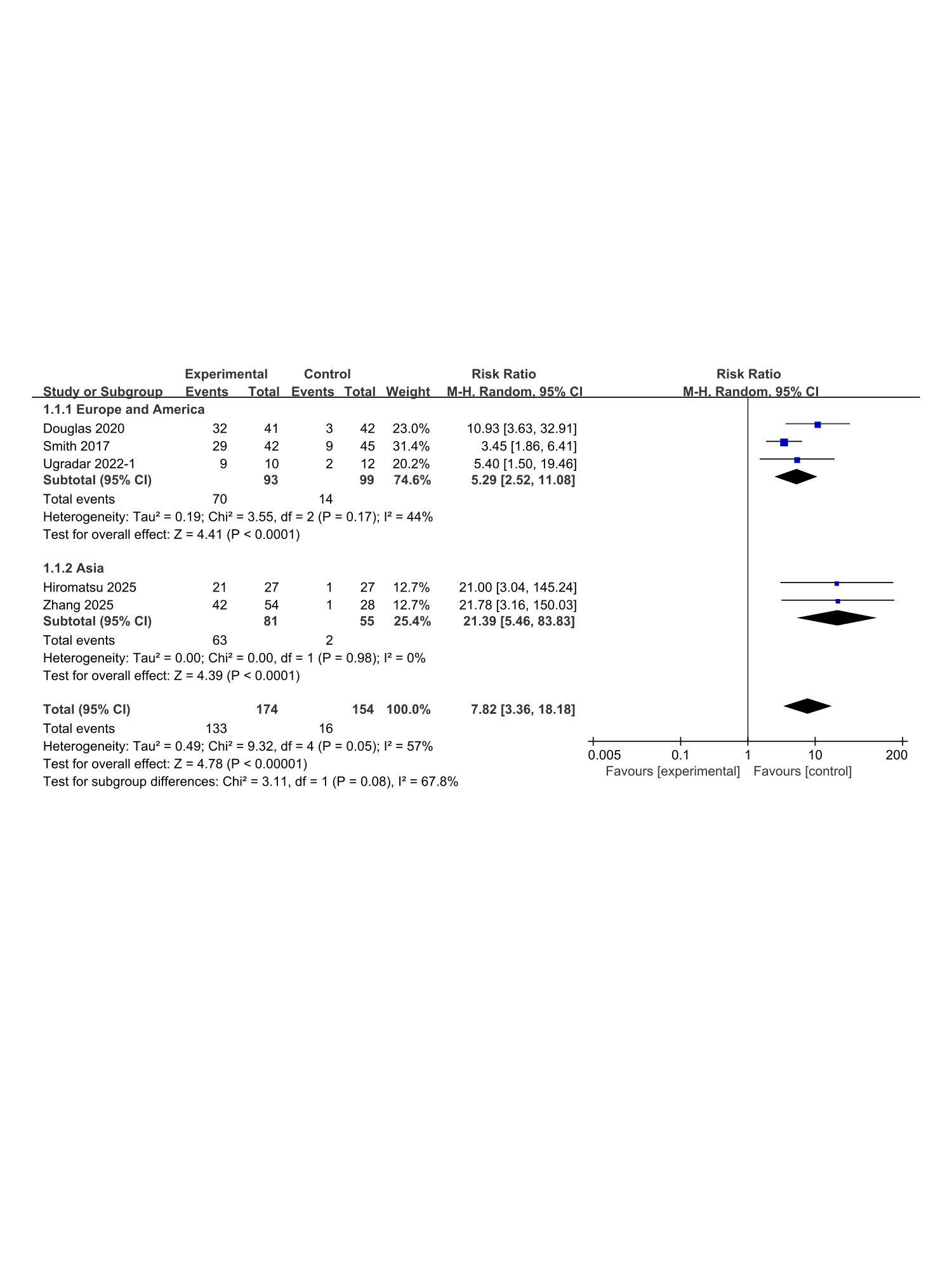

Supplement: Supplementary Figure 3 — Change in proptosis from the baseline comparing teprotumumab and placebo. [file Image3.jpeg]

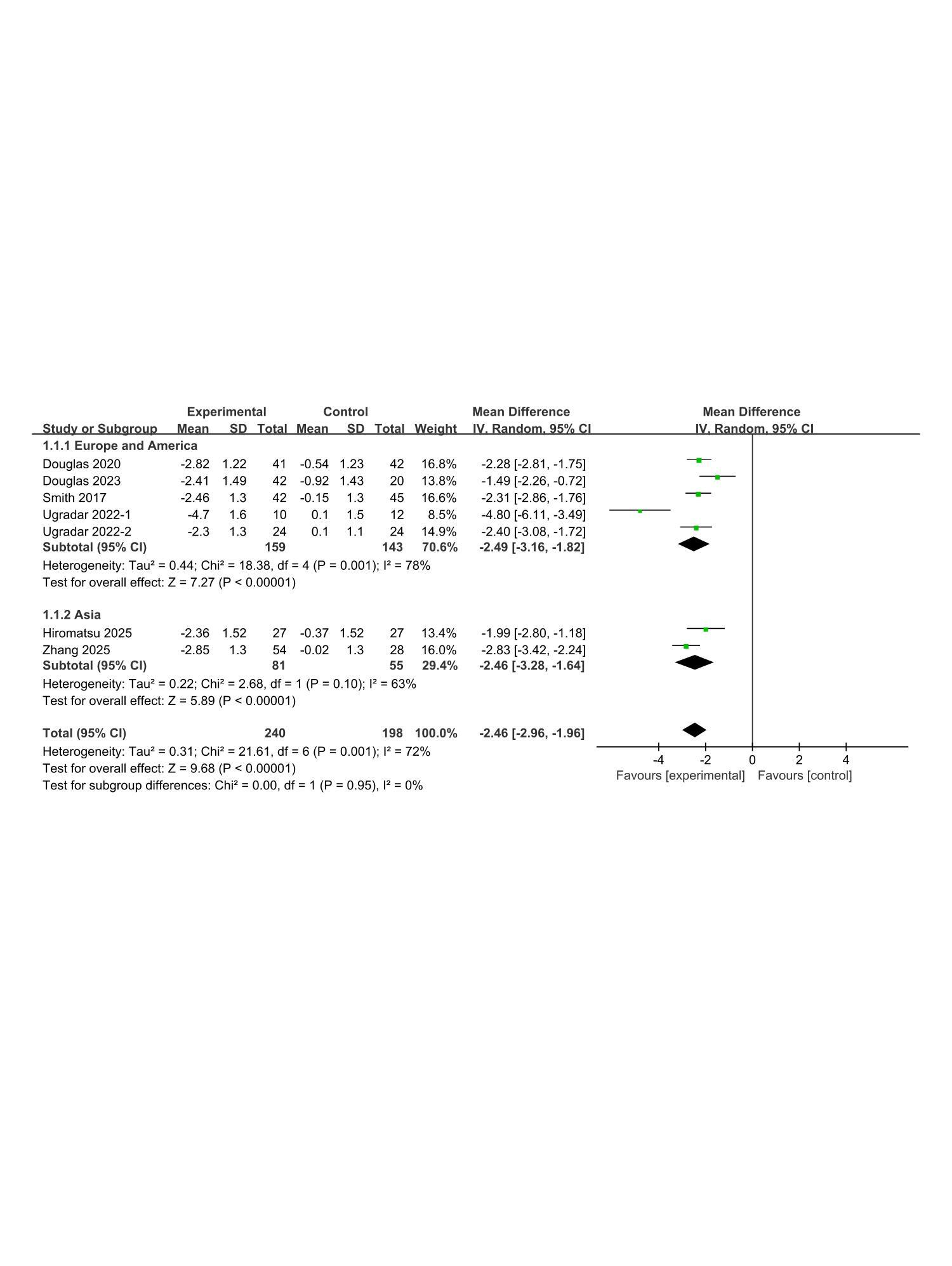

Supplement: Supplementary Figure 4 — Diplopia response rate comparing teprotumumab and placebo. [file Image4.jpeg]

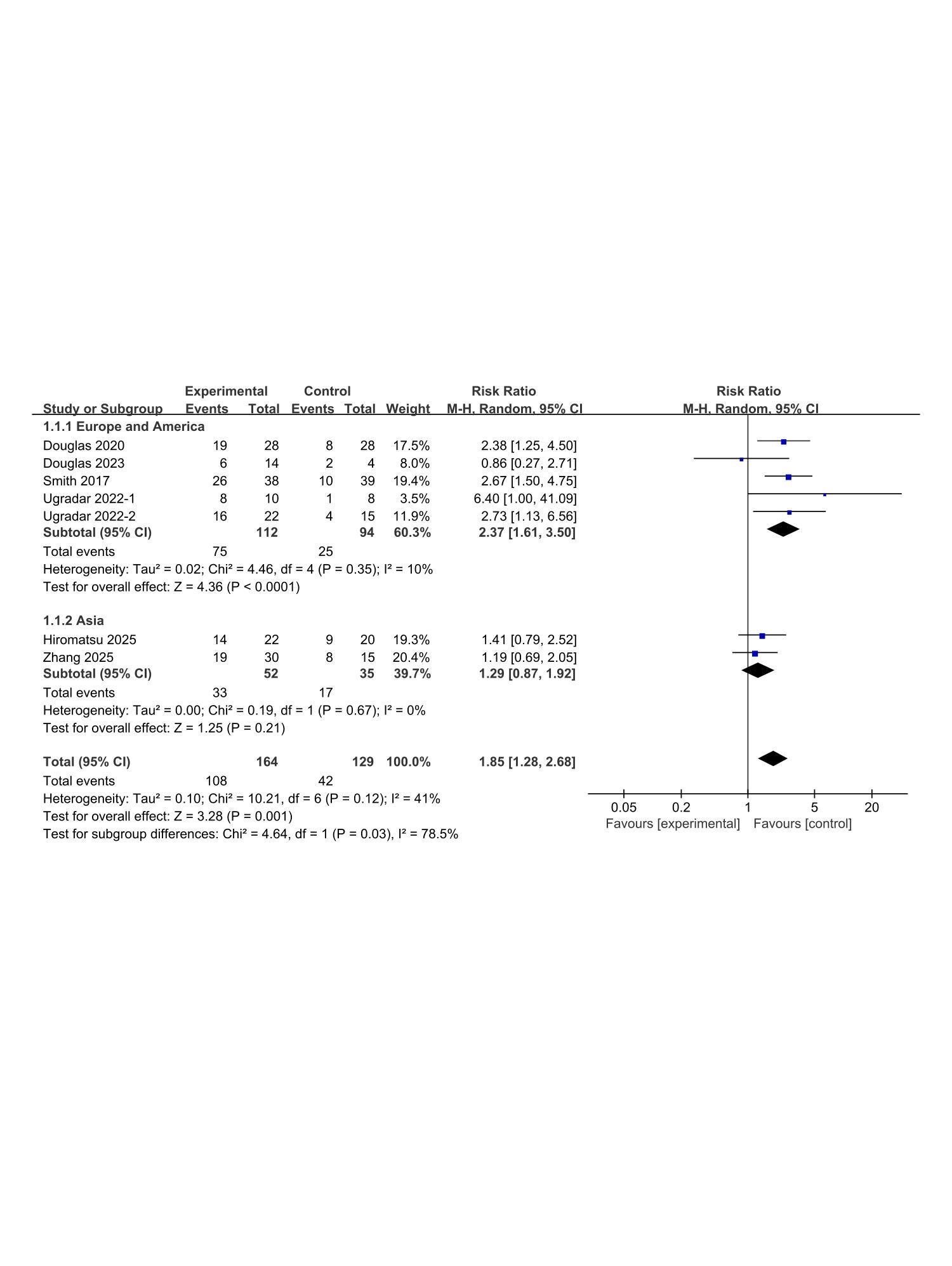

Supplement: Supplementary Figure 5 — Proportion of subjects with a clinical activity score ≤1 comparing teprotumumab and placebo. [file Image5.jpeg]

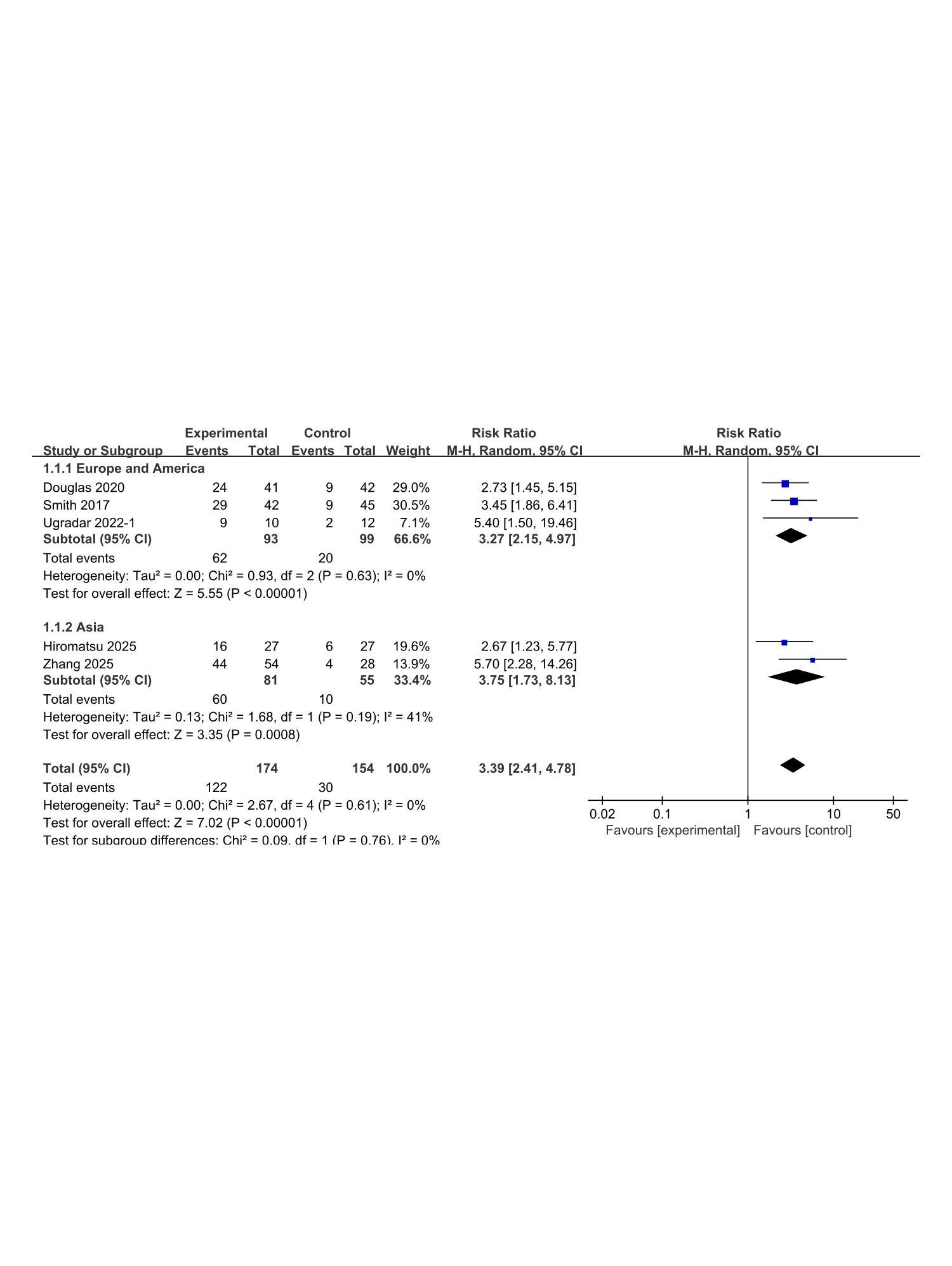

Supplement: Supplementary Figure 6 — Change in GO-QOL score from baseline comparing teprotumumab and placebo. [file Image6.jpeg]

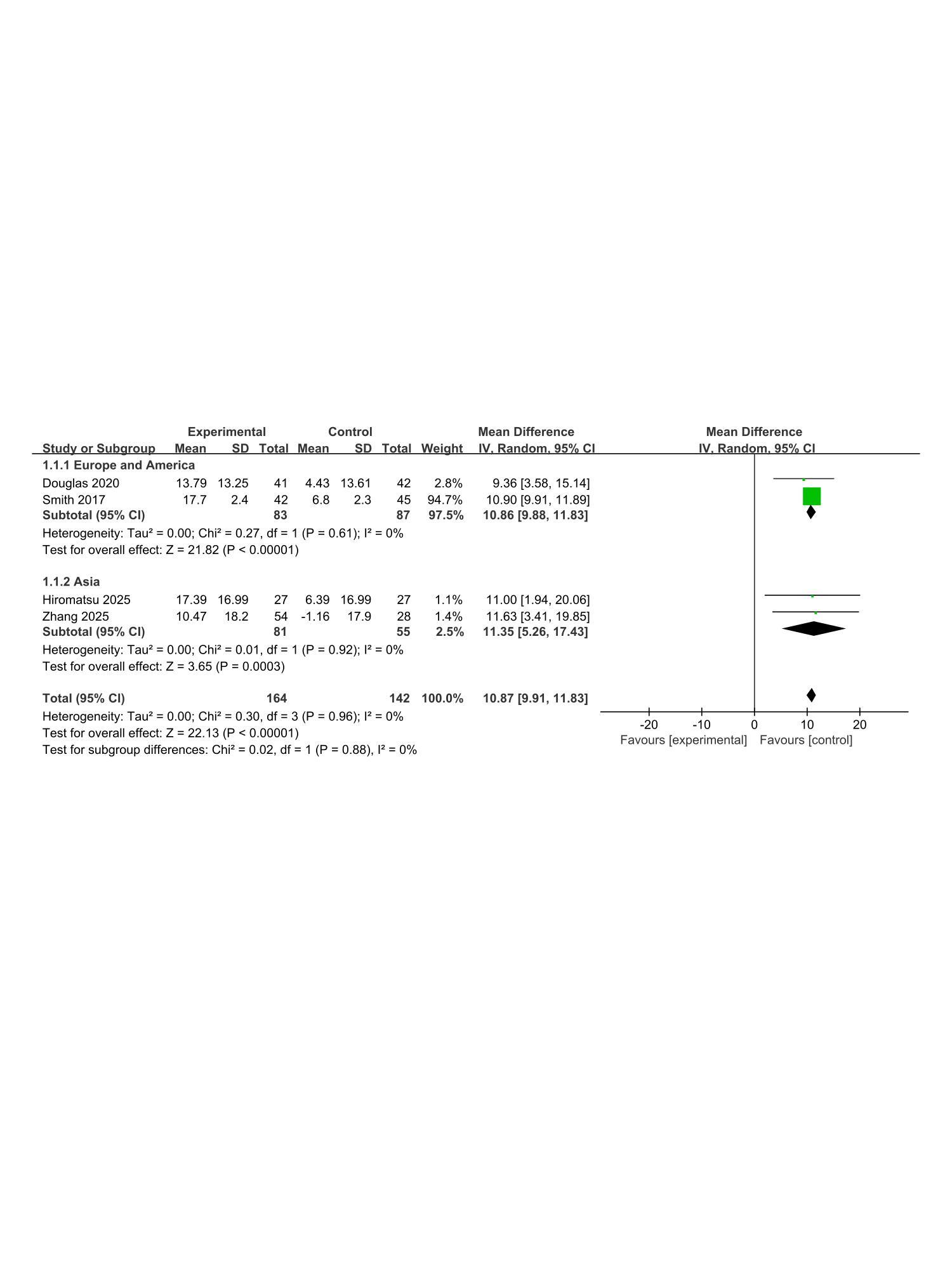

Supplement: Supplementary file 7 [file Image7.jpeg]
