# Supplementary material for: Redefining evidence for teprotumumab in thyroid eye disease: an updated meta-analysis of efficacy and safety
Source: Front Endocrinol (Lausanne). 2026 Feb 13;17:1735660. doi: 10.3389/fendo.2026.1735660 (PMC12945760; doi:10.3389/fendo.2026.1735660)
Supplement: Supplementary file 8 [file Table1.docx]

**Supplementary table S1. Search strategy**

**PubMed**

#1 "Graves Ophthalmopathy"[Mesh] 3694

#2  (((((((((((((((((((((((((((((((((((Graves’ orbitopathy[Title/Abstract]) OR (thyroid associated orbitopathy[Title/Abstract])) OR (thyroid eye disease[Title/Abstract])) OR (Graves’ ophthalmopathy[Title/Abstract])) OR (Ophthalmopathy, Graves[Title/Abstract])) OR (Graves Orbitopathy[Title/Abstract])) OR (Orbitopathy, Graves[Title/Abstract])) OR (Ophthalmopathies, Thyroid-Associated[Title/Abstract])) OR (Thyroid-Associated Ophthalmopathies[Title/Abstract])) OR (Thyroid Associated Ophthalmopathies[Title/Abstract])) OR (Ophthalmopathy, Thyroid-Associated[Title/Abstract])) OR (Ophthalmopathy, Thyroid Associated[Title/Abstract])) OR (Thyroid-Associated Ophthalmopathy[Title/Abstract])) OR (Thyroid Associated Ophthalmopathy[Title/Abstract])) OR (Dysthyroid Ophthalmopathy[Title/Abstract])) OR (Dysthyroid Ophthalmopathies[Title/Abstract])) OR (Ophthalmopathy, Dysthyroid[Title/Abstract])) OR (Graves Eye Disease[Title/Abstract])) OR (Disease, Graves Eye[Title/Abstract])) OR (Eye Disease, Graves[Title/Abstract])) OR (Thyroid Eye Disease[Title/Abstract])) OR (Disease, Thyroid Eye[Title/Abstract])) OR (Eye Disease, Thyroid[Title/Abstract])) OR (Thyroid Eye Diseases[Title/Abstract])) OR (Congestive Ophthalmopathy[Title/Abstract])) OR (Congestive Ophthalmopathies[Title/Abstract])) OR (Ophthalmopathy, Congestive[Title/Abstract])) OR (Ophthalmopathy, Infiltrative[Title/Abstract])) OR (Infiltrative Ophthalmopathies[Title/Abstract])) OR (Infiltrative Ophthalmopathy[Title/Abstract])) OR (Edematous Ophthalmopathy[Title/Abstract])) OR (Edematous Ophthalmopathies[Title/Abstract])) OR (Ophthalmopathy, Edematous[Title/Abstract])) OR (Myopathic Ophthalmopathy[Title/Abstract])) OR (Myopathic Ophthalmopathies[Title/Abstract])) OR (Ophthalmopathy, Myopathic[Title/Abstract]) 8130

#3 #1 OR #2 8616

#4 "teprotumumab" [Supplementary Concept] 213

#5 (((((((((((((R 1507[Title/Abstract]) OR (R-1507 monoclonal antibody[Title/Abstract])) OR (R-1507[Title/Abstract])) OR (R1507[Title/Abstract])) OR (RO-4858696-000[Title/Abstract])) OR (RO4858696-000[Title/Abstract])) OR (RO4858696[Title/Abstract])) OR (RO-4858696[Title/Abstract])) OR (RO-4858696000[Title/Abstract])) OR (RV-001[Title/Abstract])) OR (RV001[Title/Abstract])) OR (tepezza[Title/Abstract])) OR (teprotumumab-trbw[Title/Abstract])) OR (IBI311[Title/Abstract]) 58

#6 #4 OR #5 241

#7 (randomized controlled trial[pt] OR controlled clinical trial[pt] OR clinical trials as topic[mesh:noexp] OR trial[ti] OR random*[tiab] OR placebo*[tiab]) 2173846

#8 #3 AND #6 AND #7 49

**EMBASE**

#1 'endocrine ophthalmopathy'/exp 8407

#2 'ophthalmopathy, graves':ab,ti OR 'graves orbitopathy':ab,ti OR 'orbitopathy, graves':ab,ti OR 'ophthalmopathies, thyroid-associated':ab,ti OR 'thyroid-associated ophthalmopathies':ab,ti OR 'thyroid associated ophthalmopathies':ab,ti OR 'ophthalmopathy, thyroid-associated':ab,ti OR 'ophthalmopathy, thyroid associated':ab,ti OR 'thyroid-associated ophthalmopathy':ab,ti OR 'dysthyroid ophthalmopathy':ab,ti OR 'dysthyroid ophthalmopathies':ab,ti OR 'ophthalmopathy, dysthyroid':ab,ti OR 'graves eye disease':ab,ti OR 'disease, graves eye':ab,ti OR 'eye disease, graves':ab,ti OR 'thyroid eye disease':ab,ti OR 'disease, thyroid eye':ab,ti OR 'eye disease, thyroid':ab,ti OR 'thyroid eye diseases':ab,ti OR 'congestive ophthalmopathy':ab,ti OR 'congestive ophthalmopathies':ab,ti OR 'ophthalmopathy, congestive':ab,ti OR 'ophthalmopathy, infiltrative':ab,ti OR 'infiltrative ophthalmopathies':ab,ti OR 'infiltrative ophthalmopathy':ab,ti OR 'edematous ophthalmopathy':ab,ti OR 'edematous ophthalmopathies':ab,ti OR 'ophthalmopathy, edematous':ab,ti OR 'myopathic ophthalmopathy':ab,ti OR 'myopathic ophthalmopathies':ab,ti OR 'ophthalmopathy, myopathic':ab,ti OR 'thyroid associated ophthalmopathy':ab,ti OR 'graves ophthalmopathy':ab,ti OR **'**thyroid associated orbitopathy':ab,ti 8033

#3 #1 OR #2 10224

#4 'teprotumumab'/exp 693

#5 'r 1507':ab,ti OR 'r-1507 monoclonal antibody':ab,ti OR 'r-1507':ab,ti OR 'r1507':ab,ti OR 'ro-4858696-000':ab,ti OR 'ro4858696-000':ab,ti OR 'ro4858696':ab,ti OR 'ro-4858696':ab,ti OR 'ro-4858696000':ab,ti OR 'rv-001':ab,ti OR 'rv001':ab,ti OR 'tepezza':ab,ti OR 'teprotumumab-trbw':ab,ti OR 'ibi311':ab,ti 127

#6 #4 OR #5 771

#7 'controlled clinical trial'/exp OR random*:ti,ab OR placebo*:ti,ab OR trial:ti 3068471

#8 [embase]/lim 34039725

#9 #7 AND #8 2343894

#10 #3 AND #6 AND #9 98

**CENTRAL (Cochrane Central Register of Controlled Trials)**

#1 MeSH descriptor: [Graves Ophthalmopathy] explode all trees 211

#2 (Ophthalmopathy, Graves OR Graves Orbitopathy OR Orbitopathy, Graves OR Ophthalmopathies, Thyroid-Associated OR Thyroid-Associated Ophthalmopathies OR Thyroid Associated Ophthalmopathies OR Ophthalmopathy, Thyroid-Associated OR Ophthalmopathy, Thyroid Associated OR Thyroid-Associated Ophthalmopathy OR Thyroid Associated Ophthalmopathy OR Dysthyroid Ophthalmopathy OR Dysthyroid Ophthalmopathies OR Ophthalmopathy, Dysthyroid OR Graves Eye Disease OR Disease, Graves Eye OR Eye Disease, Graves OR Thyroid Eye Disease OR Disease, Thyroid Eye OR Eye Disease, Thyroid OR Thyroid Eye Diseases OR Congestive Ophthalmopathy OR Congestive Ophthalmopathies OR Ophthalmopathy, Congestive OR Ophthalmopathy, Infiltrative OR Infiltrative Ophthalmopathies OR Infiltrative Ophthalmopathy OR Edematous Ophthalmopathy OR Edematous Ophthalmopathies OR Ophthalmopathy, Edematous OR Myopathic Ophthalmopathy OR Myopathic Ophthalmopathies OR Ophthalmopathy, Myopathic):ti,ab,kw 696

#3 #1 OR #2 696

#4 (R 1507 OR R-1507 monoclonal antibody OR R-1507 OR R1507 OR RO4858696 OR RO-4858696 OR RO-4858696000 OR RV-001 OR RV001 OR tepezza OR teprotumumab-trbw OR IBI311 OR teprotumumab):ti,ab,kw 97

#5 #3 AND #4 64
